# Legionella control in the water system of antiquated hospital buildings by shock and continuous hyperchlorination: 5 years experience

**DOI:** 10.1186/1471-2334-14-394

**Published:** 2014-07-16

**Authors:** Giovanni Battista Orsi, Matteo Vitali, Lucia Marinelli, Veronica Ciorba, Daniela Tufi, Angela Del Cimmuto, Paolo Ursillo, Massimo Fabiani, Susi De Santis, Carmela Protano, Carolina Marzuillo, Maria De Giusti

**Affiliations:** 1 Department of Public Health and Infectious Diseases, “Sapienza” University of Rome, Piazzale Aldo Moro 5, 00185 Rome, Italy

**Keywords:** *Legionella* spp, Infection control, Hyperchlorination, Environmental monitoring

## Abstract

**Background:**

To control the presence of *Legionella* in an old hospital water system, an integrated strategy of water disinfection-filtration was implemented in the university hospital Umberto I in Rome.

**Methods:**

Due to antiquated buildings, hospital water system design and hospital extension (38 buildings), shock hyperchlorination (sodium hypochlorite, 20–50 ppm of free chlorine at distal points for 1–2 h) followed by continuous hyperchlorination (0.5-1.0 mg/L at distal points) were adopted, and microbiological and chemical monitoring of the water supply was carried out in the university hospital (December 2006-December 2011).

**Results:**

Overall, 1308 samples of cold <20°C (44.5%), mixed ≥20°C ≤ 45°C (37.7%) and hot >45°C (17.8%) water were collected, determining residual free chlorine (0.43 ± 0.44 mg/L), pH (7.43 ± 0.29) and trihalomethanes (8.97 ± 18.56 μg/L). *Legionella* was isolated in 102 (9.8%) out of 1.041 water samples without filters (*L. pneumophila* sg 1 17.6%, *L. pneumophila* sg 2–14 28.4%, *L.* non *pneumophila* 53.9%), and in none of the 267 samples with filters. *Legionella* was recovered in 23 buildings out of 38 and 29 samples (28.4%) exceeded 10^3^ cfu/L. When considering the disinfection treatment *Legionella* was isolated: before shock hyperchlorination (21.1%), 15 days after shock hyperchlorination (7.8%), 30 days after shock hyperchlorination (3.5%), during continuous hyperchlorination (5.5%) and without continuous hyperchlorination (27.3%). Continuous hyperchlorination following the shock treatment achieved >70% reduction of positive samples, whereas no continuous hyperchlorination after shock treatment was more frequently associated to *Legionella* isolation (OR 6.41; 95% CI 3.10–13.26; p <0.001). Independent risk factors for *Legionella* isolation were: residual free chlorine <0.5 mg/L (OR 13.0; 95% CI 1.37 – 123.2; p <0.03), water T° ≥20°C ≤ 45°C (OR 12.0; 95% CI 1.28 – 111.48; p <0.03) and no continuous hyperchlorination after shock treatment (OR 10.3; 95% CI 1.06 – 100.05; p <0.05).

**Conclusions:**

Shock and continuous hyperchlorination achieved significant *Legionella* reduction, but effective chlorine levels (>0.5 < 1.0 mg/L) deteriorated water quality (organoleptic and chemical). However, shock and continuous hyperchlorination remains a valid-term option in old buildings with no water system rational design, managing problems due to hospital extension and absence of a proper hot water recirculation system.

## Background

Over the past 30 years, our understanding of the reservoir and ecology of *Legionella* spp. has increased substantially. Factors that most enhance colonization of water systems include water temperature, presence of dead branches, obstruction and stagnation of the water flow, pipes material, corrosion, biofilm formation in plumbing network and the presence of other microrganisms such as protozoa that support the growth of *Legionella* spp. [1-4].

Biofilm accumulation favours the proliferation of algae and protozoa which provide *Legionella* with essential nutrients and interfere with the action of disinfectants. Also antiquated plumbing materials adopted support microbial growth and the development of biofilms. A very important single factor contributing to increased *Legionella* population is represented by dead-ends in the water distribution system because they are not reached by the disinfection treatments. Furthermore in large hospitals it may be difficult to maintain hot water at stable levels as *Legionella* flourishes at temperatures between 20°C-50°C. All these risk factors for *Legionella* colonization are often present in water distribution system of antiquated buildings. Therefore old hospitals represent a major problem for *Legionella* prevention because many risk factors for its growth are usually present [5].

Although hospitals aim is to guarantee the absence of *Legionella* from their water distribution systems, there is no ideal method for ensuring total disinfection, and it is accepted that eliminating *Legionella,* once it has colonized a water supply, is extremely difficult [6-10]. Furthermore hospital antiquated buildings with conditions favouring biofilm proliferation, very often add structural and technical limits to the selection of an appropriate disinfection method [1,4].

Specific data are needed to assist hospitals with antiquated buildings in making a decision regarding the purchase of a disinfection system for controlling *Legionella* in the water system, specially in an outbreak situation when remedial action must be undertaken at very short notice [3,11]. Superheat is an emergency measure frequently adopted but it may not be applicable in some large old hospitals where hot water recirculation is missing. Therefore, in these cases, if immediate measures are needed shock disinfection is the only option [8], even if chemicals may present several disadvantages. In particular sodium hypochlorite is easier to use than other chemical treatments that present some management difficulties that will be described in the following discussion.

Following two cases of hospital legionellosis occurred between December 2006 and January 2007 [12] in the university hospital Umberto I in Rome, in order to prevent and control the presence of *Legionella* in the hospital water system, a special program was implemented.

The study describes the results of a five-year monitoring program applied to the water distribution system of the hospital, in order to evaluate the efficacy of an integrated disinfection-filtration strategy in controlling *Legionella* spp. colonization of the hospital water system.

## Methods

**Figure 1:**
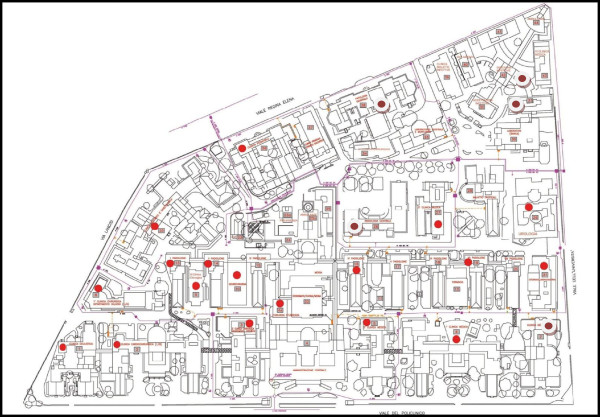
Umberto I University Hospital buildings where Legionella was isolated from water samples.

**Figure 2:**
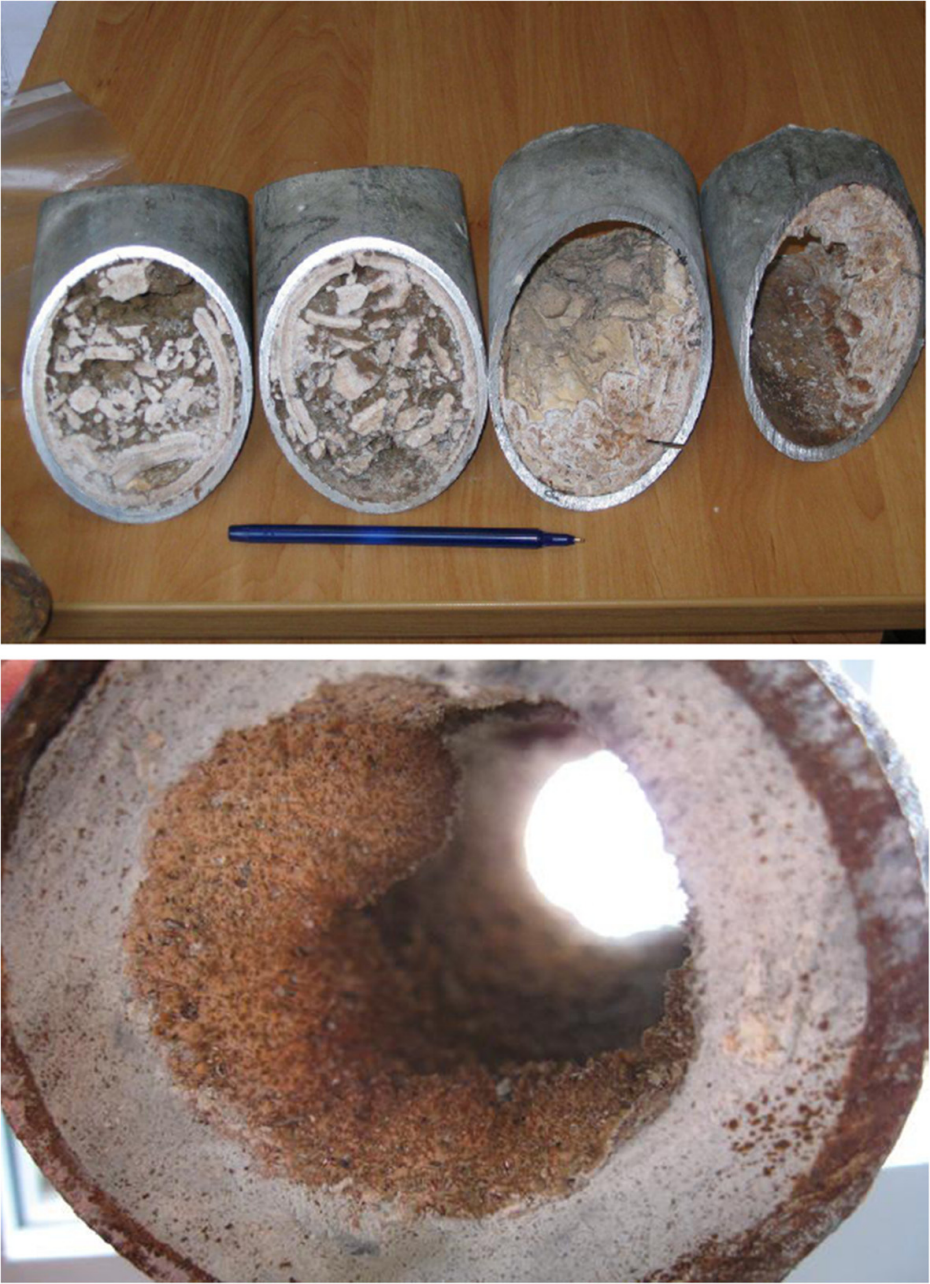
Inorganic deposits and biofilm in some of the water system tubes.

The university hospital Umberto I of Rome (1,200 beds), founded in 1893, extends over 13 hectares and most of the 38 buildings were built in the first half of the 20th century (Figure 1). The water plumbing system is very complex, partially antiquated (outdated pipes material, corrosion, presence of dead branches, limited water recirculation…) (Figure 2) and without a complete maintenance register.

As the hospital extends over a large surface area, the water supply is divided between a central network providing water to every building and a secondary independent water distribution system with one or more large boilers serving hot and cold water to the different units within the block (i.e. administration offices, general wards, nurseries, intensive care units…). Over the years there have been major renovations of the central water supply network, whereas the buildings secondary distribution systems have received mainly emergency repairs with the extension of some water lines which has created new dead branches.

Overall, the university hospital has no hot water recirculation system and the cold water network does not distribute the water rationally. All these conditions represent a relevant risk factor for the growth of *Legionella* bacteria.

Due to the antiquated buildings, design of water system, lack of a hot water circuit and extent (38 buildings) of the hospital, various treatment options such as superheating, chlorine dioxide, copper-silver ionization and monochloramine were excluded by the hospital management. Thus, a chlorine supply system was installed at the entrance of the secondary independent water distribution system of each building. In all buildings, a shock hyperchlorination (sodium hypochlorite, 20–50 ppm of free chlorine at distal points for 1–2 h) was carried out, followed by continuous hyperchlorination (0.5-1.0 mg/L) performed only in those buildings where patients were present. On the contrary there was no continuous chlorination in a few buildings without patients. We monitored the disinfection procedures from December 2006 to December 2011 [8,13].

Chlorine was added to the cold water at the entrance of the secondary independent water distribution system of each building. Free chlorine was determined again in the water leaving the boilers by an automatic system and eventually, if necessary, more chlorine was added.

No antifilm-forming substances were added to the water for fears of patient safety.

In addition, in high risk units (intensive care units, haemathology, neonatology, transplant unit), point of use filters were installed on water taps and replaced every 30 days according to the manufacturer’s specifications (Filtranios 30 LPA, polietersulfone 0,2 μm, Laboratories ANIOS France). There were periodic inspections, cleaning and maintenance of the water distribution systems, decalcification and/or replacement of showers/taps. Simultaneously, there was routine microbiological surveillance and chemical monitoring of the water supply.

The surveillance plan involved a systematic monitoring of the water system in all 38 buildings based on:

a) definition of 120 remote sampling points, selected for each building on a specific risk assessment, including the distribution system, the distance from the chlorine pump, water temperature (cold, hot and mixed) and patient susceptibility to legionellosis;

b) definition of the time planning: for each building, water samples were collected before the first shock hyperchlorination, 15–30 days after the shock hyperchlorination, during continuous hyperchlorination (every six months). Legionella monitoring was also carried out in a few buildings where, after shock hyperchlorination, the chlorine supply system was not implanted as no patients were present;

c) *Legionella* spp. detection followed the methods described in the “Italian guidelines for Legionellosis prevention and control” [14,15]. Also, the drinking water microbiological parameters, according to the Italian [16] and European [17] regulations for human consumption, were monitored. The reference parameters for the water, in order to be declared potable, were the following: *Enterococcus* spp., *Escherichia coli* and *Pseudomonas aeruginosa* should not be detectable in 100 mL; total viable count (TVC) less than 100 and 20 colony forming units (cfu) per mL at 22°C and 37°C respectively.

d) physical-chemical and chemical parameter determinations associated with Legionella disinfection treatments (hardness, conductivity, temperature, residual disinfectant concentrations, pH, trihalomethanes)

### Water sampling and *Legionella* analysis

According to Italian and European regulations [16,17], all samples were collected, without flaming and flushing for one minute, in sterile bottles with sodium thiosulphate to neutralize any residual chlorine, and immediately transported in a cool box (2°C-6°C) to the laboratory.

Only viable planktonic *Legionella* bacteria were counted [14,15]. One litre was filtered using 0.2 μm isopore polycarbonate membranes (Millipore Corporation, Bedford, MA, USA); these were then resuspended in 10 mL of the same water sample and vortexed: 5 mL was treated at 50°C for 30’ and seeded (0.1 mL) on glycine vancomycin polymixin B cycloheximide medium (GVPC, Abtek Biologicals Ltd, Liverpool, UK). The remaining 5 mL was cold seeded using the same technique. After incubation at 36°C for 8–10 days in a humified environment at 2.5% CO_2_, the plates were evaluated every day for a maximum of 14 days, in order to detect suspect *Legionella* colonies and quantitative assessment was made and expressed in cfu/L [18]. The suspect colonies were subcultered on buffered charcoal yeast extract (BCYE) agar with cysteine and charcoal yeast extract agar (CYE Agar Base), and those ascribable to the *Legionella* genus were serologically identified by the agglutination Legionella Latex test (Oxoid, Ltd, Basingstoke, UK and Biomerieux France) which provides separate identification of *Legionella pneumophila* serogroup 1, *L. pneumophila* serogroup 2–14 and species of *Legionella* spp. according to Dresden monoclonal antibody typing [19].

### Water sampling and chemical analysis

pH, temperature, residual chlorine, hardness and conductivity were determined according to the Italian reference method [20]. In particular, for the measurement of trihalometanes, 25 mL of each sample were injected in a purge & trap apparatus (TeledyneTekmar, Philadelphia, USA), extracted in the vapour phase by helium bubbling, concentrated in a specific retaining trap and directly injected in the gas-chromatography which was coupled to a single quadruple mass spectrometer (Agilent, Santa Clara, USA).

### Data analysis

Statistical analyses were performed using Epi-Info (version 2011; CDC, Atlanta, GA). The chi-square test was used to examine differences between groups. Statistical significance was defined as a P value of less than 0.05. Univariate relationships were tested using odds ratios and 95% confidence intervals (CI_95_). Logistic regression analysis was also used to adjust for potential confounders.

The association between chlorination stage and Legionella isolation was evaluated considering the following parameters:

a) Before hyperchlorination (20–50 ppm of free chlorine) vs. 15 days after hyperchlorination (20–50 ppm of free chlorine);

b) Before hyperchlorination (20–50 ppm of free chlorine) vs. 30 days after hyperchlorination (20–50 ppm of free chlorine);

c) Before hyperchlorination (20–50 ppm of free chlorine) vs. Continuous hyperchlorination (0.5-1.0 mg/L of free chlorine);

d) Continuous hyperchlorination (0.5-1.0 mg/L of free chlorine) vs. 30 days after hyperchlorination (20–50 ppm of free chlorine);

e) No continuous hyperchlorination (0.5-1.0 mg/L of free chlorine) vs. Continuous hyperchlorination (0.5-1.0 mg/L of free chlorine);

Multivariate analysis was used to estimate the effects of risk factors and potential confounders on Legionella isolation. For this purpose we used a stepwise logistic regression technique; the model was constructed using Legionella isolation (coded as No = 1 and Yes = 0) as the dependent variable, and water temperature (≥20°C - ≤45°C = 1 and <20°C or > 45°C = 2), residual free chlorine concentration (<0.5 = 1 and ≥ 0.5 = 2), potability of water (not potable water = 1 and potable water = 2), no continuous vs. continuous hyperchlorination (no continuous hyperchlorination = 1 and continuous hyperchlorination = 2) as the independent variables. The level of significance for inclusion was set at 0.05.

The results of multivariate analysis, include potability of the water even though the effect of this factor is not statistically significant at the 5% level. The reason for including potability is that this variable is of fundamental interest for the interpretation of the analysis.

## Results

From December 2006 until December 2011, 38 buildings were included and overall 1,308 samples of water were analyzed for the presence of *Legionella*, whereas a smaller number of samples (404 samples), only from cold and mixed water, were also screened for all other microbial drinking water parameters. With regard to the chemical parameters, chlorine was determined in all the 1,308 water samples, whereas trihalomethanes in 310. Average water pH was 7.43 ± 0.29 and remained constant during hyperchlorination. Water samples were represented by cold < 20°C (15.4°C ± 2.0°C) 44.5%, mixed ≥ 20°C ≤ 45°C (33.4°C ± 8.0°C) 37.7% and hot > 45°C (51.4°C ± 3.8°C) 17.8% water. Table 1 shows residual free chlorine (0.43 ± 0.44 mg/L) and trihalomethanes (8.97 ± 18.56 μg/L) levels in relation to the chlorination stage.

**Table 1:** Levels of free residual chlorine, trihalomethanes and Legionella isolation according to chlorination stage

**Chlorination stage**	**Residual free chlorine mg/L**				**Trihalomethanes μg/L**				**Legionella spp. Samples without point of use filters**		**Legionella spp. Samples with point of use filters**	
	**Arithmetic mean**	**SD**	**Median**	**IQ Range**	**Arithmetic mean**	**SD**	**Median**	**IQ Range**	**Positive**	**Total**	**Positive**	**Total**
**Before hyperchlorination**	0.19	0.56	0.10	0.05 - 0.20	2.71	1.81	2.38	1.36 - 3.17	43 (21.1%)	204	0	4
**15 days after hyperchlorination**	0.34	0.73	0.18	0.10 - 0.25	8.70	6.61	7.50	3.95 - 12.5	15 (7.8%)	193	0	18
**30 days after hyperchlorination**	0.23	0.18	0.20	0.11 - 0.30	8.55	3.52	7.84	6.38 - 11.30	6 (3.5%)	173	0	20
**Continuous hyperchlorination**	0.61	0.34	0.60	0.40 - 0.79	10.7	23.8	5.50	2.76 - 10.01	23 (5.5%)	416	0	222
**No continuous hyperchlorination**	0.15	0.15	0.12	0.07 - 0.17	1.05	1.07	0.88	0.66 - 2.62	15 (27.8%)	55	0	3
**Total**	**0.43**	**0.44**	**0.30**	**0.13 - 0.65**	**8.97**	**18.56**	**5.40**	**2.58 - 9.87**	**102 (9.8%)**	**1041**	**0**	**267**

The drinking water bacteriological parameters were generally within the expected values, in accordance with the Italian [16] and European [17] regulations.

In 23 buildings out of 38 (Figure 1) *Legionella* was isolated in 102 (9.8%) samples of water without filters (Table 1). Twenty-nine samples (28.4%) exceeded 10^3^ cfu/L (limit value for Italian guideline), and 32.3% of positive samples presented concentrations of *Legionella* between 10^2^ and 10^3^ cfu/L (threshold level for intervention according to Italian guideline) [18]. Table 1 shows the number of positive samples for *Legionella* according to the chlorination treatment stage. Remarkably: 1) as expected no *Legionella* or other monitored bacteria was recovered in samples collected from taps equipped with point of use filter; 2) continuous hyperchlorination after shock treatment achieved >70% reduction of samples positive for *Legionella* in water distribution system.

Serotyping performed on isolates revealed that *L. pneumophila* sg 1 were 17.6%, *L. pneumophila* sg 2–14 28.4% and remaining *Legionella* spp 53.9% (including one *L. anisa* isolate). *Legionella* serotypes and isolated concentrations in water are reported in Table 2. During the study no significant association was found between buildings colonization and specific serogroups.

**Table 2:** Distribution of Legionella species by serogroup and concentration (cfu/L)

**Legionella serotype**	**< 102 UFC/L**	**≥ 102 < 103 UFC/L**	**≥ 103 < 104 UFC/L**	**≥ 104 UFC/L**	**Total**
**Legionella pneumophlia sg. 1**	4 (22.2%)	8 (44.4%)	6 (33.3%)	0	18 (17.6%)
**Legionella pneumophila sg. 2-14**	2 (6.9%)	10 (34.5%)	11 (37.9%)	6 (20,7%)	29 (28.4%)
**Legionella other species**	34 (61.8%)	15 (27.3%)	5 (9.1%)	1 (1.8%)	55 (53.9%)
**Total**	**40 (39.2%)**	**33 (32.3%)**	**22 (21.6%)**	**7 (6.9%)**	**102**

**Figure 3:**
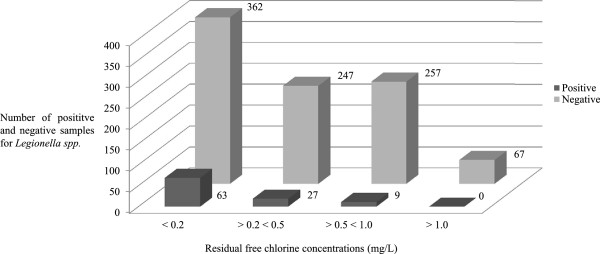
Number of positive and negative samples for Legionella spp. according to residual free chlorine concentrations (mg/L).

Results of univariate analyses evidenced that *Legionella* isolation was significantly associated to no continuous chlorination after shock hyperchlorination treatment (OR 6.41; 95% CI 3.10–13.26; p <0.001). (Tables 3 and 4). As expected, free chlorine concentrations resulted lower in samples positive for *Legionella* by comparison to negative ones (0.32 ± 0.30 vs. 0.61 ± 0.36; p < 0,001) (Figure 3). It was noticed that *Legionella* isolation was more frequently isolated from mixed water samples (9.1%) than from cold (7.5%) or hot (3.5%) water samples.

**Table 3:** Distribution of Legionella spp. by concentration (cfu/L) according to chlorination stages

	**< 102 cfu/L**	**> 102 < 103 cfu/L**	**> 103 cfu/L**	**Total**
**Before hyperchlorination**	9 (4.4%)	21 (10.3%)	13 (6.4%)	43/204 (21.1%)
**15 days after hyperchlorination**	8 (4.1%)	2 (1.0%)	5 (2.6%)	15/193 (7.8%)
**30 days after hyperchlorination**	0	4 (2.3%)	2 (1.2%)	6/173 (3.5%)
**Continuous hyperchlorination**	8 (1.9%)	5 (1.2%)	10 (2.4%)	23/416 (5.5%)
**No continuous hyperchlorination**	13 (23.6%)	2 (3.6%)	0	15/55 (27.3%)*
**Total**	**38**	**34**	**30**	**102**

*Odds Ratio = 6.41, 95% CI 3.10 - 13.26; p < 0.001 no continuous hyperchlorination compared with continuous hyperchlorination.

**Table 4:** Risk factors associated with Legionella isolation in univariate analysis

**Risk factors**	**Parameters**	**Legionella isolation no. (%)**				
		**Yes**	**No**	**OR**	**95% CI**	**p-value**
**Water temperature°C**	≥20°C - ≤ 45°C	48 (13.3)	314 (86.7)	1.98	1.30 - 3.03	< 0.001
vs.	<20°C or > 45°C	47 (7.2)	609 (92.8)			
**Residual free chlorine concentration mg/L**	< 0.5	90 (12.9)	610 (87.1)	5.3	2.63 - 10.65	< 0.001
vs.	≥ 0.5	9 (2.7)	323 (97.3)			
**Potability of water according to regulation**	Not potable water	9 (25.0)	27 (75.0)	4.48	1.87 - 10.74	0.001
vs.	Potable water	21 (6.9)	282 (93.1)			
**No continuous vs. continuous hyperchlorination**	No continuous hyperchlorination	15 (27.3)	40 (72.7)	6.41	3.10 - 13.26	< 0.001
vs.	Continuous hyperchlorination	23 (5.5)	393 (94.5)			
**Before vs. shock hyperchlorination at 15 days**	Before shock hyperchlorination	43 (21.1)	161 (78.9)	3.17	1.70 - 5.92	< 0.001
vs.	Shock hyperchlorination at 15 days	15 (7.8)	178 (92.2)			
**Before vs. shock hyperchlorination at 30 days**	Before shock hyperchlorination	43 (21.1)	161 (78.9)	7.43	3.08 - 17.94	< 0.001
vs.	Shock hyperchlorination at 30 days	6 (3.5)	167 (96.5)			
**Before vs. continuous hyperchlorination**	Before shock hyperchlorination	43 (21.1)	161 (78.9)	4.56	2.66 - 7.82	< 0.001
vs.	Continuous hyperchlorination	23 (5.5)	393 (94.5)			
**Continuous vs. shock hyperchlorination at 30 days**	Continuous hyperchlorination	23 (5.5)	393 (94.5)	1.63	0.65 - 4.07	0.15
vs.	Shock hyperchlorination at 30 days	6 (3.5)	167 (96.5)			

Multivariate analysis was carried out considering *Legionella* isolation as dependent variable and free chlorine < 0.5 mg/L, 20 ≤ T° ≤ 45°C, no continuous hyperchlorination, non-compliance with drinking water indicators as independent variables was carried out. The results showed that independent risk factors for *Legionella* isolation were residual free chlorine <0.5 mg/L (OR 13.0; 95% CI 1.37 – 123.2; p <0.03), water T° ≥20°C ≤ 45°C (OR 12.0; 95% CI 1.28 – 111.48; p <0.03) and no continuous hyperchlorination after shock treatment (OR 10.3; 95% CI 1.06 – 100.05; p <0.05) (Table 5).

**Table 5:** Risk factors associated to Legionella isolation in multivariate logistic regression analysis

**Risk factors**	**Parameters**	**OR**	**95% CI**	**p-value**
**Water temperature°C**	≥20°C - ≤45°C	11.96	1.28 - 111.48	0.0293
	<20°C or > 45°C			
**Residual free chlorine concentration mg/L**	< 0.5	12.97	1.37 - 123.17	0.0256
	≥ 0.5			
**Potability of water according to regulation**	Not potable water	3.57	0.77 - 16.65	0.1052
	Potable water			
**No continuous vs. continuous hyperchlorination**	No continuous hyperchlorination	10.30	1.06 - 100.05	0.0444
	Continuous hyperchlorination			

## Discussion

As experienced by others [3,8] the two cases of hospital legionellosis which occurred at the university hospital Umberto I generated media negative publicity and malpractice suits, requesting immediate measures to minimize panic among patients and employees. Unfortunately the hospital design and structure, multiple buildings, large extension, aged plumbing system, lack of hot water circuit obliged the hospital management to exclude various treatment options.

Superheating is an emergency measure frequently adopted but was not applicable in the absence of a hot water circuit system. Chlorine dioxide and monochloramine are effective in reducing *Legionella* water colonization, yelding low chlorine concentrations, but they require more time, equipment and maintenance than chlorination. Copper-silver ionization is recognized to be effective although as ion concentrations monitoring system is needed, but at the time it was not adoptable in an emergency condition. Moreover, according to the European Commission Decision 2012/78/UE copper compounds actually are not included in the list of disinfectants to be used for water disinfection. Hydrogen peroxide with silver ions at the time was still experimental [1,3,7,21,22].

Therefore, in order to rapidly control *Legionella* in the water system, immediate shock and continuous hyperchlorination were adopted in most hospital buildings [8,13]. In addition, inspection, cleaning, water distribution systems maintenance, decalcification and/or replacement of showers/taps were provided, and point of use filters on water taps were installed and replaced every 30 days in high risk units.

European and Italian guidelines recommend an increase of clinical and environmental surveillance when *Legionella* spp. exceed the threshold level of 10^3^ cfu/L [13,14,23], whereas the Allegheny County guidelines relate infection risk to the proportion of water sites contaminated by *Legionella* spp. rather than to the bacterial concentration and suggest disinfection when the proportion of positive sites is >30% [24]. Remarkably, during the five-year study, our risk management plan was successful in reducing significantly both *Legionella* concentration and proportion of positive water samples in the university hospital water system.

Overall after water shock and continuous hyperchlorination the sample points positive for *Legionella* spp. decreased significantly (P < 0.05) from 21.1% to 5.5% with a reduction of >70% (Table 1). Moreover the samples exceeding 10^3^ cfu/L decreased from 6.4% to 2.4% with a reduction of >60% (P < 0.05). Drinking water microbial contamination was low and no association with legionella was found.

It is well known that, after the initial reduction induced by the shock treatment, the colonization tends to recur after some weeks often at even higher levels [11,25]. Results highlighted that effective continuous hyperchlorination after shock treatment was decisive in achieving *Legionella* reduction, whereas low level free chlorine was the most important independent risk factor associated to legionella isolation (Table 3). Another important independent risk factor associated to Legionella isolation was water temperature ≥20°C ≤ 45°C.

As precedingly reported in the methods, first shock hyperchlorination (20–50 ppm of free chlorine) was carried out in all hospital buildings, but later continuous hyperchlorination (0.5-1.0 mg/L) was not applied in a few buildings without patients.

The treatment did not succeed in eradicating completely *Legionella* spp. contamination, as the microorganism was still detectable at concentrations >10^3^ cfu/L in 2.4% of the samples examined. These results are in agreement with previous reports indicating that *Legionella* spp. may persist in hospital environment for several years, even at undetectable levels as Legionella Viable but non Culturable (VBNC), causing sporadic infections or epidemics [26,27]. Even if hyperchlorination of the water is consistently performed, this approach is particularly appropriate for the treatment and removal of planktonic cultures of legionella, but remains ineffective against sessile communities of the bacterium [28,29].

In our experience, continuous free chlorine levels between 0.5 and 1.0 mg/L (Figure 3) were effective in reducing significantly *Legionella* presence in the old hospital water system. Unfortunately the continuous hyperchlorination at >0.5 < 1.0 mg/L determined the non potability of drinking water [16], and the production of disinfectant by-products such as trihalomethanes increased. Therefore in some hospital special units (i.e. dialysis, neonatal unit…) it was necessary to implement special equipment in order to reduce the high levels of free chlorine and to eliminate the trihalomethanes. In consideration of the hospital water continuous hyperchlorination at >0.5 < 1.0 mg/L, all patients received mineral water and in the bathrooms a specific notice was reported. However only nine samples (2.9%) out of 310 exceeded the Italian regulation limit (30 μg per litre) for trihalomethanes.

Only point of use filter achieved 100% negative samples (Table 1), but the high costs limited a wide hospital application which was confined only to high risk wards [11,30].

## Conclusions

In a large hospital with antiquated buildings, shock and continuous hyperchlorination achieved significant *Legionella* reduction, but did not eliminate it completely. Over time, the continuous hyperchlorination resulted more important than the single shock hyperchlorination treatment to limit the presence of *Legionella*. The point of use filters were very efficacious, but their use was limited to the high risk units because of cost. The absence of a proper hot water recirculation system limited enormously the treatment options adoptable in an emergency condition, however our experience could be very useful for others in similar conditions.

Continuous hyperchlorination treatments performed at effective levels (>0.5 < 1.0 mg/L) can deteriorate water quality (both organoleptic and chemical characteristics).

The *Legionella* risk assessment process should consider the criterion of *Legionella* spp. concentrations and the extent of contamination: however the control process could be improved by screening the prevalent clones virulence by molecular characterization of the isolates. Also, any prevention strategy for *Legionella* spp. in hospital water system should include active surveillance of legionellosis.

## Competing interests

The authors declare that they have no competing interests.

## Authors’ contributions

GBO MV and MDG conceived the study, analysed the data and contributed to the writing. VC, PU, CP collected and analysed the data. LM, CP contributed to the writing. LM, DT, ADC, MF, SDS, CM isolated the strains and drafted the manuscript. All authors read and approved the final manuscript.

## Pre-publication history

The pre-publication history for this paper can be accessed here:

http://www.biomedcentral.com/1471-2334/14/394/prepub
